# Alzheimer's Disease Dementia as the Diagnosis Best Supported by the Cerebrospinal Fluid Biomarkers: Difference in Cut-Off Levels from Thai Experience

**DOI:** 10.1155/2012/212063

**Published:** 2012-07-16

**Authors:** V. Senanarong, N. Siwasariyanon, L. Washirutmangkur, N. Poungvarin, C. Ratanabunakit, N. Aoonkaew, S. Udomphanthurak

**Affiliations:** Division of Neurology, Faculty of Medicine Siriraj Hospital, Mahidol University, Bangkok 10700, Thailand

## Abstract

*Objectives*. To determine how beta-amyloid 1-42 (A**β** 1-42), total tau (tTau), and phosphorylated tau (pTau) levels in CSF behave in a cohort of Thai patients from the Memory Clinic in Bangkok, Thailand. *Methods*. During 2009–2011, twenty eight subjects from the memory clinic at Siriraj Hospital had CSF analysis for AD biomarkers. A**β** 1-42, tTau, and pTau (at amino acid 181) were measured in CSF by ELISA technique. *Results*. Mean of Thai mental state examination (TMSE) of 28 Thai cohort was 16.48 (6.63). Fourteen had AD, ten had non-AD dementia, and four non-cases were those with subjective memory complaint (SMC) without dementia. Mean CSF A**β** 1-42, tTau, ptau (181), and pTau/A**β** 1-42 in the AD group were 241.36 (60.14) pg/mL, 222.79 (212.24) pg/mL, 40.79 (27.84) pg/mL, and 0.18 (0.12) accordingly. Mean CSF A**β** 1-42, tTau, pTau (181), and pTau/A**β** 1-42 in the non-AD dementia group were 430.40 (125.18) pg/mL, 349.30 (692.16) pg/mL, 36.80 (14.90) pg/mL, and 0.09 (0.04) accordingly. Mean CSF A**β** 1-42, tTau, pTau (181), and pTau/A**β** 1-42 in the non-cases with SMC without dementia were 499.75 (93.44) pg/mL, 137.25 (62.74) pg/mL, 31.75 (17.48) pg/mL, and 0.06 (0.02). There is significant difference (*P* < 0.05) among the 3 groups in CSF A**β** 1-42 and pTau/A**β** 1-42. We propose mean + 1.5 SD of CSF A**β** 1-42 in AD group (331.57 pg/mL) to be the cut-off point in Thai subjects. *Conclusion*. There are significant different in CSF A**β** 1-42 and CSF p-tau/A**β** 1-42 among those with AD, non-AD dementia and non cases with SMC without dementia in Thai cohort. Cut-off point of CSF A**β** 1-42 of 331.57 pg/mL is suggested in Thai study.

## 1. Introduction

 The Hallmark pathology of Alzheimer's disease (AD) is senile plaques (SPs) and  neurofibrillary tangles (NFTs) [1]. The principal component of the SP is the hydrophobic beta amyloid (A*β* 1-42). The hyperphosphorylated tau (pTau) is a characteristic component of NFT. The pTau is a fraction of concentrated total Tau (tTau) protein in the NFT. Previous studies demonstrated that cerebrospinal fluid biomarkers of A*β* 1-42, tTau, and pTau (at amino acid 181) can differentiate individuals with AD from healthy controls with a good sensitivity and specificity, but cut-off levels differ between test centers [2, 3]. Culturally, acceptance of cerebrospinal fluid (CSF) biomarkers as part of dementia investigation varies from regions to regions. Ageism is still a burden of dementia diagnosis. Comorbidity with vascular risk factors and previous infection in the central nervous system are important in developing countries. We set up CSF biomarkers for dementia for 4 years at the Memory Clinic at Siriraj Hospital and started a service to individual with subjective memory complaint (SMC) with or without dementia in the past 3 years. Offering lumbar puncture to SMC individuals with gait problems is acceptable to Thai patients and caregivers. In addition to look for CSF biomarker for dementia, routine CSF analysis to rule out inflammation and infection is done as well. Because of public awareness of unusual dementia problems in early age, lumbar puncture as dementia working diagnosis is acceptable in Thai population point of view. Earlier investigation showed no difference in CSF biomarkers between early onset dementia (EOD) of Alzheimer's disease and late onset of Alzhiemer's disease (LOAD) [4]. 

## 2. Objectives

We aimed to compare CSF levels of A*β* 1-42, total tau (tTau), and phosphorylated tau at threonine 181 (pTau 181) between AD, non-AD dementia, and noncases. We hope to look at possible new cut-off point of CSF biomarkers in our Thai population cohort.

## 3. Methods

 During 2009–2011, the Memory Clinic in Bangkok at Siriraj Hospital set up the investigations of cerebrospinal fluid biomarkers for the service in cases with unusual manifestation to the clinic. Twenty eight subjects had CSF analysis for AD biomarkers. CSF was obtained by lumbar puncture between the L3/L4 or L4/L5 intervertebral space, using a 25-gauge needle, and collected in 10 mL polypropylene tubes. Within 2-3 hours, CSF samples were centrifuged at 2,100 g for 10 minutes at 4°C. One milliliter of CSF was used for routine analysis. Routine CSF analysis was also done to look for infection or metastasis. Aliquots of each sample were immediately frozen at minus 80°C until further analysis. A*β* 1-42, tTau, and pTau (at amino acid 181) were measured in CSF by enzyme-linked immunoassay (ELISA) technique of the INNOTEST, Ghent, Belgium. All patients underwent a standardized clinical assessment, including medical history, physical and neurological examination including Thai Mental State Examination (TMSE, Thai version of MMSE) [5, 6], laboratory investigations, neuropsychometric evaluation, brain magnetic resonance imaging (MRI), or computerized tomography (CT). The TMSE has the range of possible scores of 0–30. The TMSE has a good correlation (*r* = −0.679, *P* < 0.001) with the Informant Questionnaire on Cognitive Decline in the Elderly (IQCODE) in an earlier study in Thai cohort [7]. The score of less than 24 is considered as dementia. Senanarong et al. [8] utilized TMSE in a countrywide cognitive survey of 3,177 Thai elderly who were 60 years old and over. The study indicated that the TMSE score of less than 12 (5th percentile) is considered as severe dementia. Final diagnosis of individuals in this cohort was made by a consensus group of multidisciplinary team of neurologists, psychiatrists, neuropsychologists, and a radiologist utilizing standard dementia criteria [9–14]. When all clinical investigations were normal, patients were considered to have subjective complaints (SMC) without dementia. SPSS 12 was used for statistical analysis. Statistical significance was defined as *P* < 0.05. Post hoc analysis of Dunnett T 3 equal variance not assumed was used. The controls in this study were defined by those without dementia but have memory problems. This study obtained approval to study from the local ethical committee at Siriraj Hospital.

## 4. Results

 Twenty eight patients with memory problems underwent CSF biomarker analysis. The consensus diagnoses revealed that fourteen had AD, ten had non-AD dementia, and four did not meet the criteria for dementia. Among 14 AD, eight were early onset dementia (EOD, age ≤ 65 years). Twelve AD presented with magnetic gait and memory problems. Two had only memory problems. Among ten non-AD dementia, two were EOD (1 CJD and 1 FTD). The diagnoses of non-AD dementia were 5 FTD, 2 DLB, 1 CJD, 1 VaD, and 1 NPH. Five non-AD dementia presented with abnormal gait, four with behavioral problems, and one with both behavioral problems and abnormal gait. Among four subjects with memory complaint without dementia, one had schizophrenia aged 24, one had chronic kidney disease and malignancy of the lungs, and two had memory complaint with hydrocephalus and cautious gait. Sixteen were women and twelve were men. Mean age of the study cohort was 68.39 (SD = 14.69) years old. The consensus diagnoses revealed that among 11 EOD, 8 had AD, 2 had FTD, and 1 had CJD. The Patient characteristics are shown in Table 1. There is no statistical difference in age and genders among those with AD, with non-AD dementia, and non cases. One patient (male aged 49 year-old) with non-AD had CJD whom CSF tTau was very high (2,314 pg/mL), CSF CSFpTau was relatively low (36 pg/mL), and CSF A*β* 1-42 was 343 pg/mL. Mean CSF A*β* 1-42, tTau, pTau (181), and pTau/A*β* 1-42 in the AD group were 241.36 (60.14) pg/mL, 222.79 (212.24) pg/mL, 40.79 (27.84) pg/mL, and 0.18 (0.12) accordingly. Mean CSF A*β* 1-42, tTau, pTau (181), and pTau/A*β* 1-42 in the non-AD dementia group were 430.40 (125.18) pg/mL, 349.30 (692.16) pg/mL, 36.80 (14.90) pg/mL, and 0.09 (0.04) accordingly. Mean CSF A*β* 1-42, tTau, pTau (181), and pTau/A*β* 1-42 in those non cases with SMC without dementia were 499.75 (93.44) pg/mL, 137.25 (62.74) pg/mL, 31.75 (17.48) pg/mL, and 0.06 (0.02).

**Figure 1:**
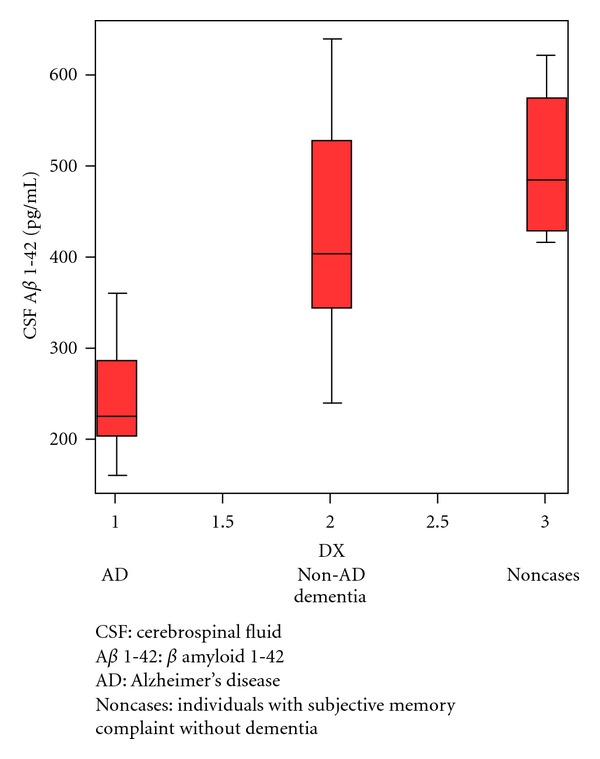
CSF A*β* 1-42 in box plot.

**Figure 2:**
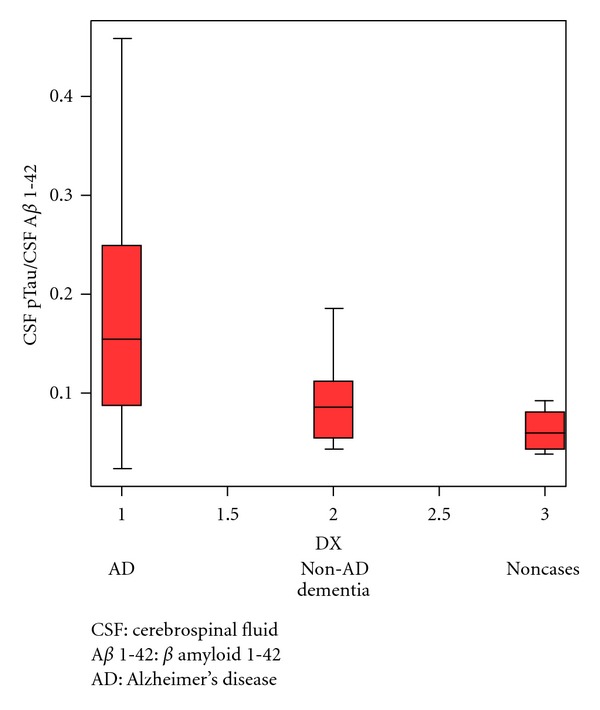
CSF pTau/CSF A*β* 1-42 in box plot.

**Figure 3:**
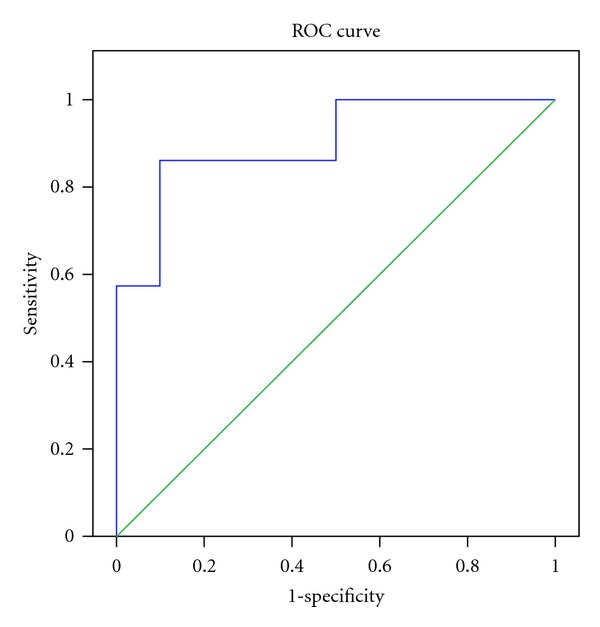
The receiver operating characteristic curve (ROC) showed mean + SD of the area under the curve (AUC) of CSF beta-amyloid 1-42 for the diagnosis of AD. (AD = 14 and non-AD dementia = 10). AUC = 0.900, SE = 0.063, *P* = 0.001*. At the cut-off level 315 pg/mL, it gives the sensitivity of 85.7% and specificity of 90%. At the cut-off level 355 pg/mL, it gives the sensitivity of 92.9% and specificity of 50%. SD: standard deviation, SE: standard errors, ROC: receiver operating characteristic curve, AD: Alzheimer's disease, *P < 0.05.

**Figure 4:**
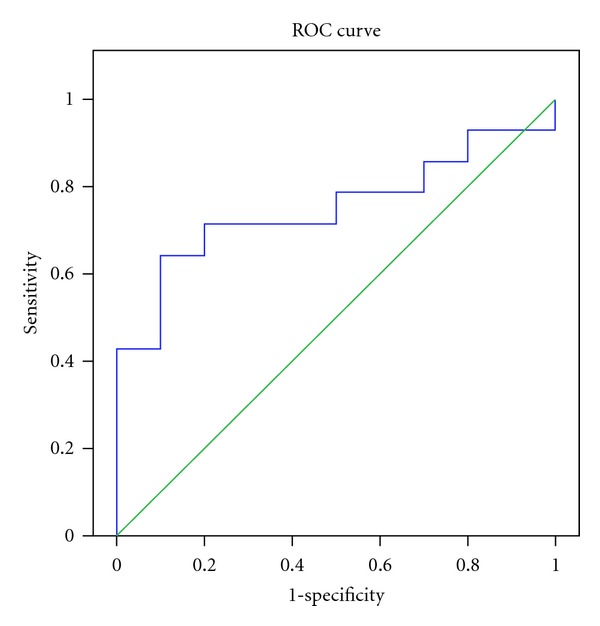
The receiver operating characteristic (ROC) curve showed mean + SD of the area under the curve (AUC) of CSF pTau/beta-amyloid 1-42 for the diagnosis of AD. (AD = 14 and non-AD dementia = 10). AUC = 0.750, SE = 0.102, *P* = 0.04*. At the cut-off level 0.1085, it gives the sensitivity of 71.4% and specificity of 70%. SD: standard deviation, SE: standard errors, ROC: receiver operating characteristic curve, AD: Alzheimer's disease,*P < 0.05.

 Our results showed that those with AD had significantly lower levels of CSF A*β* 1-42 than those with non-AD dementia and non- cases. There was no statistical difference in the level of CSF pTau and tTau among the three groups. The results of CSF pTau/CSF A*β* 1-42 also had statistical difference among the three groups (Table 2). Figures 1 and 2 showed box plot of mean levels of CSF A*β* 1-42 and CSF pTau/CSF A*β* 1-42. We proposed a cut-off levels of CSF A*β* 1-42 of less than 331.57 pg/mL (mean+1.5 SD of that from Thai AD cohort) in our laboratory investigation. When utilizing the receiver operating characteristic (ROC) curve analysis using AD (*n* = 14) as diagnosis and individual with non-AD dementia (*n* = 10) as controls, the area under the cure (AUC) of CSF A*β* 1-42 is 0.900 (SE = 0.063, *P* = 0.001*). The coordinates of the ROC curve showed that CSF A*β* 1-42 cut-off level 315 pg/mL gives the sensitivity of 85.7% and specificity of 90%, and CSF A*β* 1-42 cut-off level 355 pg/mL gives the sensitivity of 92.9% and specificity of 50%. (Figure 3) The area under the curve (AUC) of CSF pTau/CSF A*β* 1-42 is 0.750 (SE = 0.102, *P* = 0.04*) (Figure 4). From our Thai cohort, we propose 0.1085 as cut-off point of CSF pTau/CSF A*β* 1-42 ratio. Thus, it gives the sensitivity of 71.4% and specificity of 70%.

## 5. Discussion

We demonstrated that CSF biomarkers using CSF A*β* 1-42, CSF tTau, and CSF pTau were useful in diagnosis of AD. Despite our small sample size, CSF A*β* 1-42 showed significant difference among AD, non-AD, and those noncases with SMC without dementia. CSF pTau/CSF A*β* 1-42 revealed significant difference between AD and non cases but showed a trend of difference between AD and non-AD groups. The cut-off point of CSF A*β* 1-42 less than 331.57 pg/mL and CSF pTau/CSF A*β* 1-42 more than 0.1085 suggest the diagnosis of AD in Thai cohort in our laboratorywith a moderate level of accuracy.

 Previous studies showed that decreased levels of CSF A*β* 1-42 less than 487 pg/mL, increased CSF phosphorylated tau (pTau 181) more than 61 pg/mL, or increased CSF total tau more than 425 pg/mL were categorized to support the diagnosis of AD, and the opposite data was against [15, 16]. However, current review uncovers various cut-off points of CSF biomarkers from different laboratory tests [17]. CSF A*β* 1-42: amyloid fragments are plausible AD biomarkers because they represent senile plaque formation which is a hallmark pathological process in AD. However, CSF A*β* 1-42 and CSF tTau are less effective in discriminating AD from other dementias (specificities of 57% for suspected non-AD dementias) [18]. International laboratories need to make sure that the reliabilities of standard laboratory practice are well maintained. Comorbidity from age and genetic vascular vulnerable factors can be important issues to help explain the difference in levels of CSF biomarkers from center to center. Regarding the complexity of AD pathology, it is possible that combinations of individual biomarkers will provide a more accurate diagnostic and prognostic data than single marker. Decreased CSF A*β* 1-42 and increased CSF pTau proteins are the most precise and reproducible chemical diagnostics of sporadic AD. Our study had only small cohort; we then tried to exaggerate the increased pTau in CSF by manipulation of CSF pTau/CSF A*β* 1-42. The figure of of CSF pTau/CSF A*β* 1-42 closed to 0.1 suggests AD pathology. Our data showed wide SD in CSF tTau levels in non-AD group because of 1 CJD with CSF tTau more than 2,000 pg/mL. Other review supports that extremely high CSF tTau indicates CJD [19].

 Limitations of this study are small sample size and no autopsy confirmation of diagnosis. The strength of our study is the demonstration of real-life everyday practice of how CSF biomarkers assist the working diagnosis of dementia in Thai population. Further investigation of validation of current cut-off point in differentiating AD from non-AD and from control subjects is needed in Thai population. Additionally, we hope to engage to become a participant in the global or European quality control CSF standardization program in the near future.

## Figures and Tables

**Table 1:** Patient characteristics mean (SD) of 28 individuals presenting with unusual manifestation to the memory clinic.

	TMSE	Age (years)	Gender: men/women
AD (*n* = 10)	15.92 (5.20)	68.29 (12.86)	5/9
Non-AD dementia (*n* = 14)	13.75 (7.78)	70.50 (11.12)	5/5
Noncases (*n* = 4)	23.75 (3.20)	63.50 (28.41)	2/2

Total (*n* = 28)	16.48 (6.63)	68.39 (14.69)	12/16

SD: Standard deviation.

AD: Alzheimer's disease.

SMC: Subjective memory complaint.

TMSE: Thai mental state examination.

**Table 2:** Results mean (SD) of CSF A*β* 1–42, tTau, pTau, and pTau/A*β* 1–42 in 3 groups of the study.

Group/CSF	CSF A*β* 1–42 (pg/mL)	CSF tTau (pg/mL)	CSF pTau (pg/mL)	CSF pTau/A*β* 1–42
AD (*n* = 14)	241.36 (60.14)	222.79 (212.24)	40.79 (27.84)	0.1807 (0.1218)
Non-AD dementia (*n* = 10)	430.40 (125.18)	349.30 (692.16)	36.80 (14.90)	0.0914 (0.0423)
Noncases (*n* = 4)	499.75 (93.44)	137.25 (62.74)	31.75 (17.48)	0.0617 (0.0238)
*P* value (ANOVA)	*P* < 0.0001^∗^	*P* = 0.672	*P* = 0.767	*P* = 0.028^∗^
Post hoc ^∗^ *P* < 0.05	AD versus non-AD^∗^ AD versus noncases^∗^ Non-AD versus noncases	—	—	AD versus non-AD (*P* = 0.060) AD versus noncases^∗^ Non-AD versus noncases

CSF: Cerebrospinal fluid.

A*β*1-42: Beta amyloid 1-42.

AD: Alzheimer's disease.
