# Corrigendum to “Alzheimer's Disease Dementia as the Diagnosis Best Supported by the Cerebrospinal Fluid Biomarkers: Difference in Cut-Off Levels from Thai Experience”

**DOI:** 10.1155/2017/2647086

**Published:** 2017-04-02

**Authors:** Vorapun Senanarong, Nobdham Sivasariyanonda, Leatchai Wachirutmanggur, Niphon Poungvarin, Chatchawan Rattanabannakit, Nuttapol Aoonkaew, Suthipol Udompunthurak

**Affiliations:** Division of Neurology, Faculty of Medicine Siriraj Hospital, Mahidol University, Bangkok 10700, Thailand

In the article titled “Alzheimer's Disease Dementia as the Diagnosis Best Supported by the Cerebrospinal Fluid Biomarkers: Difference in Cut-Off Levels from Thai Experience” [1], the names of four authors were incorrectly spelled as “N. Siwasariyanon,” “L. Washirutmangkur,” “C. Ratanabunakit,” and “S. Udomphanthurak.” The correct spelling and the full names of authors are shown above.
